# The Overlap of Kidney Failure in Extrapulmonary Sarcoidosis in Children—Case Report and Review of Literature

**DOI:** 10.3390/ijms24087327

**Published:** 2023-04-15

**Authors:** Adriana Mocanu, Roxana Alexandra Bogos, Laura Mihaela Trandafir, Elena Cojocaru, Ileana Ioniuc, Mirabela Alecsa, Vasile Valeriu Lupu, Lucian Miron, Tudor Ilie Lazaruc, Ancuta Lupu, Ingrith Crenguta Miron, Iuliana Magdalena Starcea

**Affiliations:** 1 Faculty of General Medicine, “Grigore T. Popa” University of Medicine and Pharmacy, 16 Universitatii Street, 700115 Iasi, Romania; 2 Nephrology Division, St. Mary’s Emergency Children Hospital, 700309 Iasi, Romania

**Keywords:** extrapulmonary sarcoidosis, nephrocalcinosis, chronic kidney disease, children

## Abstract

Sarcoidosis is a non-necrotizing granulomatous inflammatory multisystemic disorder of unknown etiology. In children, as in adults, it can involve a few or all organ systems to a varying extent and degree, entailing multisystemic manifestations. Kidney involvement in pediatric-onset adult-type sarcoidosis is rare, with a wide range of renal manifestations, most of them related to calcium metabolism. Children with renal sarcoidosis tend to be more symptomatic than adults, although male patients have a higher prevalence. We present the case of a 10-year-old boy who presented with advanced renal failure with nephrocalcinosis and important hepatosplenomegaly. The diagnosis was established by histopathological examination, with consequent cortisone therapy and hemodialysis. This review emphasizes that sarcoidosis should be considered in the differential diagnosis of pediatric patients with acute kidney insufficiency or chronic kidney disease of an unknown etiology. As far as we know, this is the first study regarding extrapulmonary sarcoidosis in children from Romania.

## 1. Introduction

Sarcoidosis is a systemic granulomatous disorder characterized by the accumulation of T lymphocytes, mononuclear phagocytes, and noncaseating granulomas in almost all affected tissues and organs. The clinical phenotypes vary from single-organ, sometimes self-limited, asymptomatic disease to multi-organ involvement with high-risk manifestations. The types of pediatric sarcoidosis are classified by age into two distinct forms: early onset sarcoidosis (triad: uveitis, arthritis, and rash, mainly caused by NOD2 mutation) and pediatric-onset adult-type sarcoidosis—preferentially involving the lung and mediastinum [1]. Kidney involvement in pediatric-onset adult-type sarcoidosis is rare, and the incidence and prevalence of kidney involvement in sarcoidosis are still uncertain [1]. The kidney can be affected by the presence of histologically proven granulomas but also by calcium metabolism alteration. For example, in adults, a cohort including more than 1200 patients with pulmonary sarcoidosis found that kidney manifestations were present in 12 percent of cases [2]. In 116 cases of pediatric sarcoidosis, renal involvement was found in only 3 patients [3,4,5].

## 2. Clinical Case

**Figure 1:**
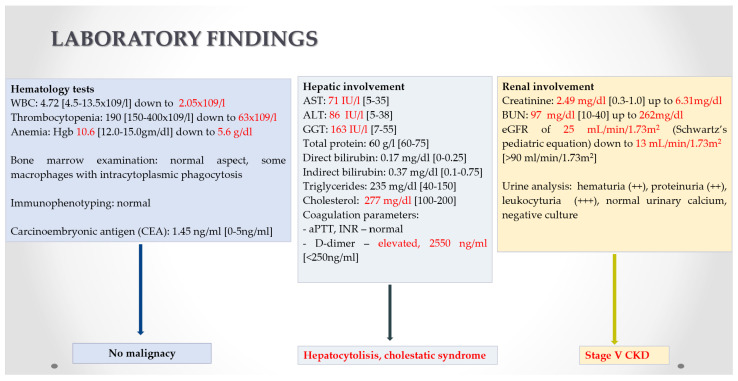
Differential diagnosis at presentation.

**Figure 2:**
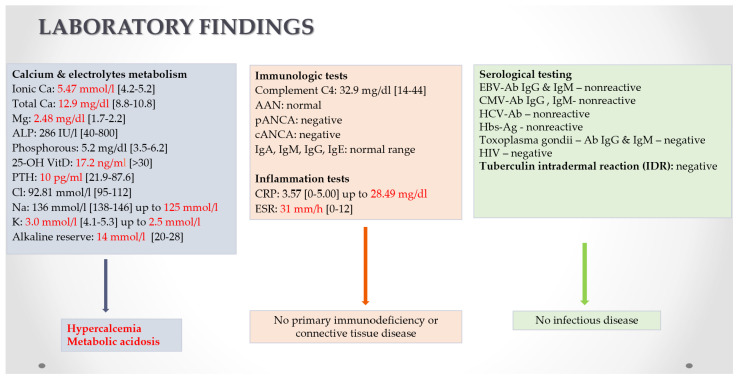
Differential diagnosis at presentation.

We illustrate the case of a 10-year-old boy who presented for weight loss (4 kg in 12 months), fatigue, and feeling sick with an insidious onset in the last year. His past medical history includes multiple respiratory infections during early childhood and nonspecific enterocolitis one year before the current presentation. On this latter occasion, hypercalcemia, as well as elevated serum urea and creatinine levels, were detected but were considered secondary to dehydration, although they did not improve with adequate hydration. Hepatic cytolysis was also constantly present in the last year. Clinical examination revealed massive hepatosplenomegaly, pallor, and oliguria, while biologically, elevated serum creatinine was noted, corresponding to an eGFR of 25 mL/min/1.73 m^2^ (Schwartz’s pediatric equation). He required admission to our pediatric nephrology department. He was hemodynamically stable, conscious, febrile, and in general distress. A follow-up evaluation revealed pancytopenia with a normal bone marrow assessment and no immunophenotyping abnormalities. Therefore, we concluded that the pancytopenia was in the liver damage. A negative carcinoembryonic antigen ruled out neoplastic disease. Moderate hepatic cytolysis and cholestasis were observed, with normal coagulation parameters. Hypercalcemia, low plasma parathyroid hormone levels, and metabolic acidosis were present, while urinalysis revealed hematuria, proteinuria, leukocyturia without hypercalciuria, and a negative urine culture. All of the differential diagnoses made upon admission to our department are summarized in Figure 1 and Figure 2.

**Figure 3:**
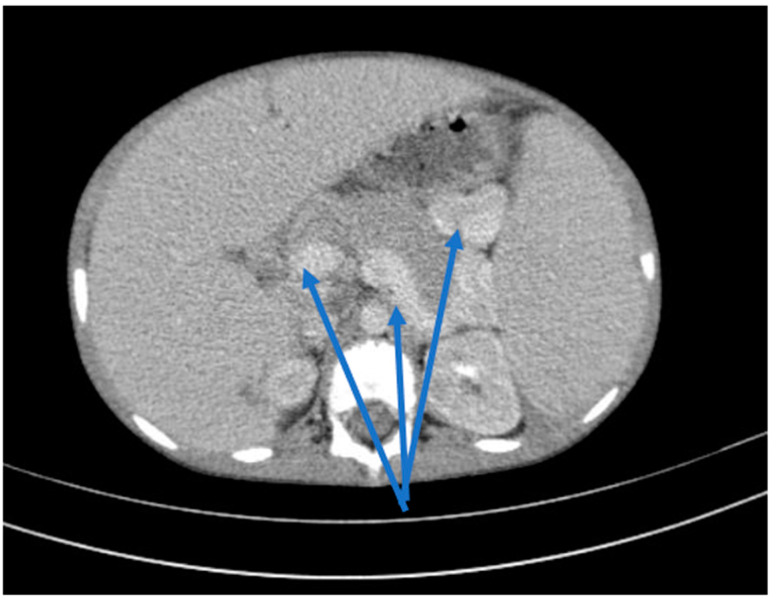
Axial CT scans, bilateral medullary nephrocalcinosis (blue arrow).

**Figure 4:**
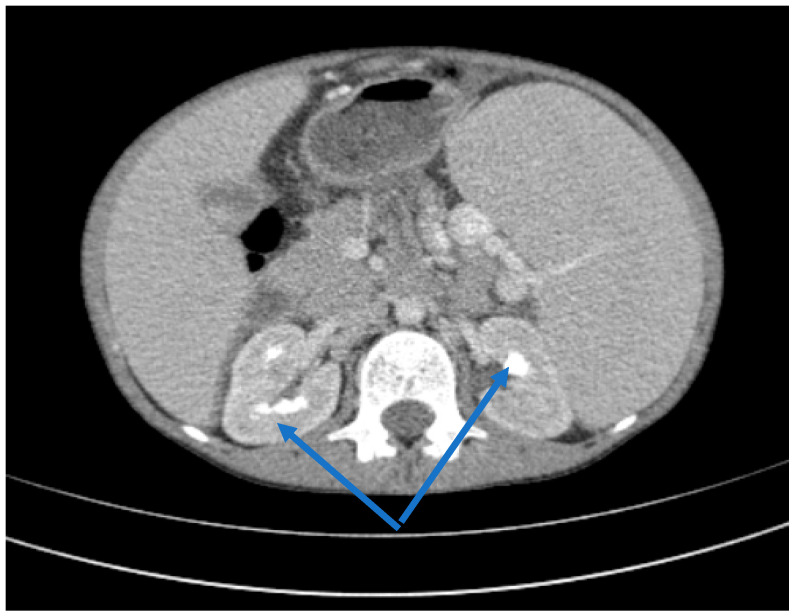
Axial CT scans, portal hypertension (blue arrow).

Renal function deteriorated rapidly to a creatinine clearance of 10 mL/min/1.73 m^2^, requiring the initiation of hemodialysis on day 5 after admission. A history of drug ingestion that could cause acute interstitial nephritis was negative. Serologic tests for Epstein–Barr virus, cytomegalovirus, hepatitis C/B, human immunodeficiency virus, *Toxoplasma gondii*, and tuberculin skin reaction were negative. In the absence of specific immunological findings, a marked inflammatory syndrome or antibodies such as p-ANCA, c-ANCA or antinuclear antibodies, primary immunodeficiency, a connective tissue disease or vasculitis were excluded. Renal ultrasound evaluation was highly suggestive of nephrocalcinosis. After this preliminary evaluation, the diagnosis of advanced renal disease was made; therefore, the patient started chronic hemodialysis. Thoracic–abdominal computed tomography confirmed renal medullary calcifications, hepatosplenomegaly, hyperdense splenorenal and splenogastric masses as indicators of portal hypertension (Figure 3 and Figure 4).

**Figure 5:**
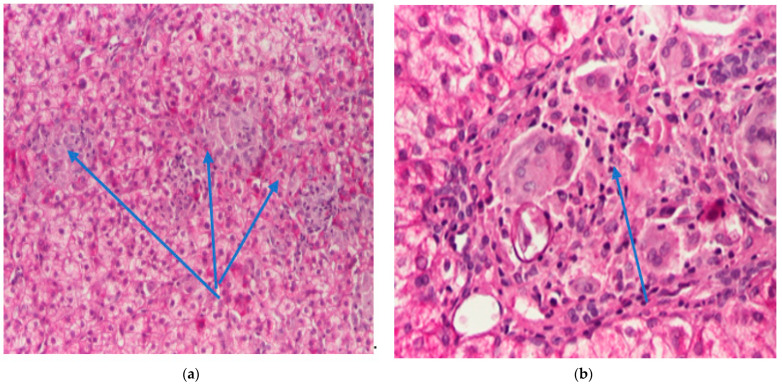
(**a**): PAS coloration ×100; (**b**): PAS coloration ×200. Liver tissue showing chronic giant cell granulomatous hepatitis (blue arrows) (personal collection, St. Mary Emergency Children’s Hospital, Iasi).

**Figure 6:**
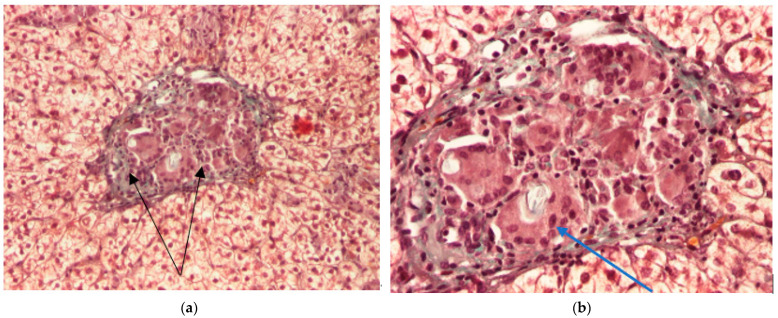
(**a**) Szekely trichrome staining ×100, (**b**) Szekely trichrome staining ×200. Liver biopsy: tight, well-formed epithelioid granuloma of sarcoidosis, not directed at the bile duct. There is a cuff of lymphocytes (black arrows) and Langhans giant cell, with the nuclei arranged in a ring along the periphery (blue arrow). Even not specific, Langhans type giant cells are characteristic of sarcoidosis (personal collection, St. Mary Emergency Children’s Hospital, Iasi).

In this context, we performed a hepatic biopsy that described chronic giant cell granulomatous hepatitis, highly evocative for sarcoidosis (Figure 5a,b and Figure 6a,b).

To support this diagnosis, serum amyloid A and serum angiotensin-converting enzyme were measured, revealing high concentrations. Peripheral band opacities resulting from calcium deposits on the Bowman’s subepithelial layer were also detected, as band keratopathy is a sarcoidosis-associated entity. Considering the renal, hepatic, and ophthalmic involvement in our patient, the diagnosis of extrapulmonary sarcoidosis was made. Our therapeutic approach was initially supportive, including the correction of hydro–electrolytic and acid-base imbalances, blood transfusion, and hemodialysis. Hepatic and portal involvement required the administration of ursodeoxycholic acid, beta-blockers, and ornithine. Specific therapy with prednisolone daily (1 mg/kg/day) was initiated, with positive results after 3 months of treatment, as presented in Table 1.

**Figure 7:**
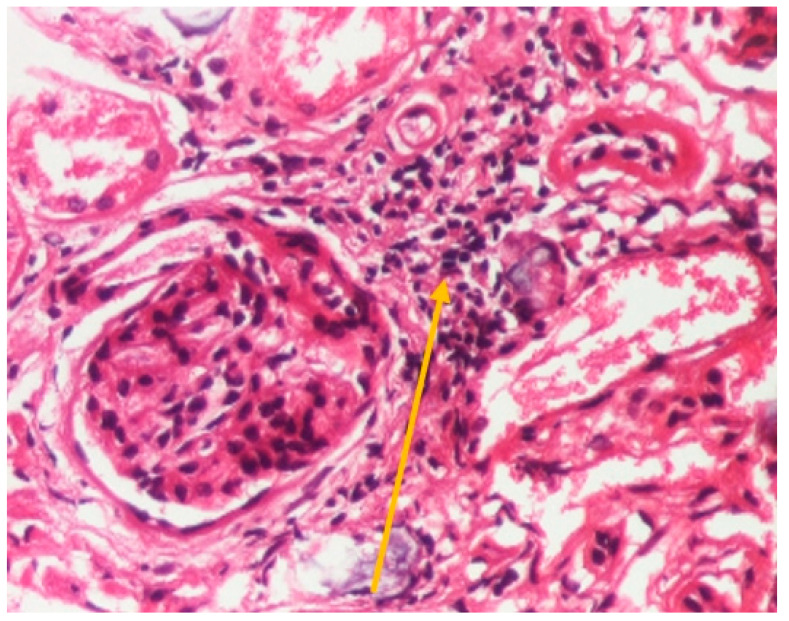
Hematoxylin-eosin staining ×200; renal tissue showing interstitial inflammation (yellow arrow) (personal collection, St. Mary Emergency Children’s Hospital, Iasi).

**Figure 8:**
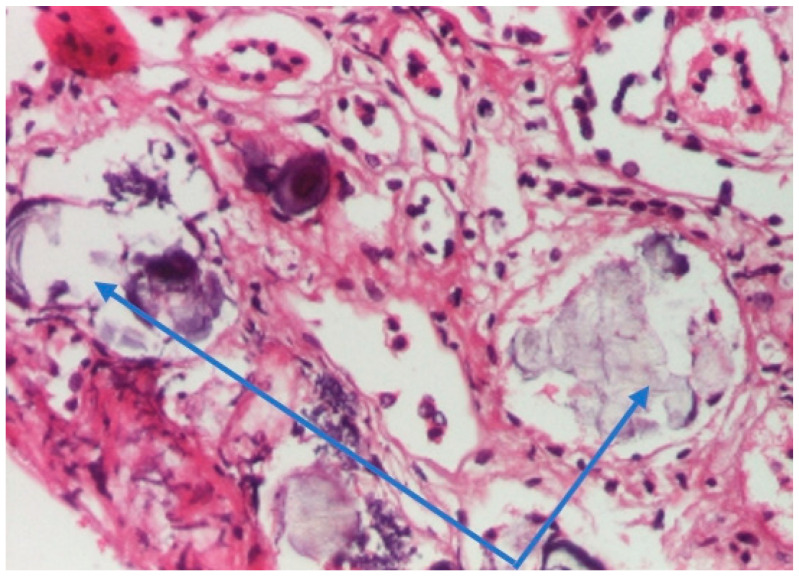
Hematoxylin-eosin staining ×200; renal tissue showing calcifications (blue arrows) (personal collection, St. Mary Emergency Children’s Hospital, Iasi).

**Figure 9:**
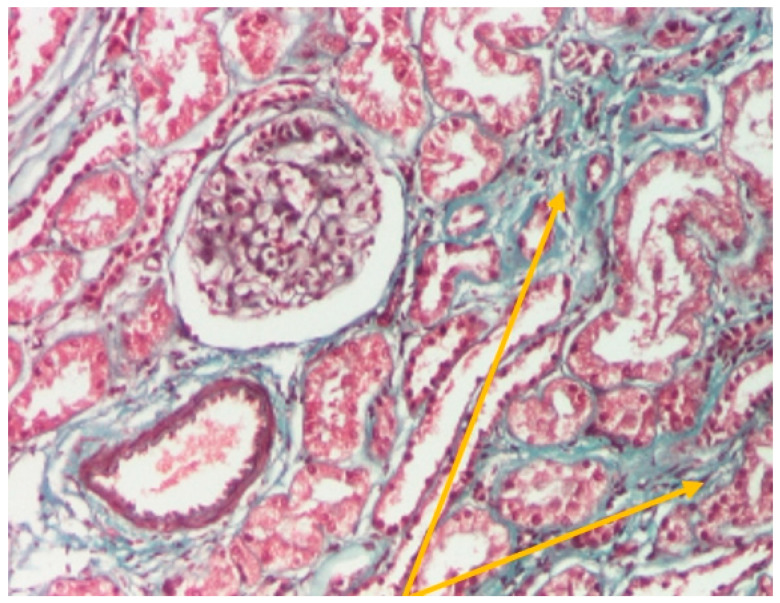
Szekely trichrome staining ×100; renal tissue showing interstitial fibrosis (yellow arrow) (personal collection, St. Mary Emergency Children’s Hospital, Iasi).

However, because of corticoid-related side effects (secondary Cushing syndrome), we decided to decrease the dose of prednisolone up to the maintenance dose of 0.25 mg/kg/day. Even though a clear histopathological diagnosis was made through hepatic punction, and renal involvement was almost certainly a consequence of sarcoidosis, this evolution prompted us to perform a renal biopsy. This showed non-granulomatous interstitial nephritis, nephrolithiasis, and interstitial fibrosis, as shown in Figure 7, Figure 8 and Figure 9. These images show no immune deposits in immunofluorescence, and no glomerulonephritis aspects, such as crescents or extent of mesangial proliferation.

**Figure 10:**
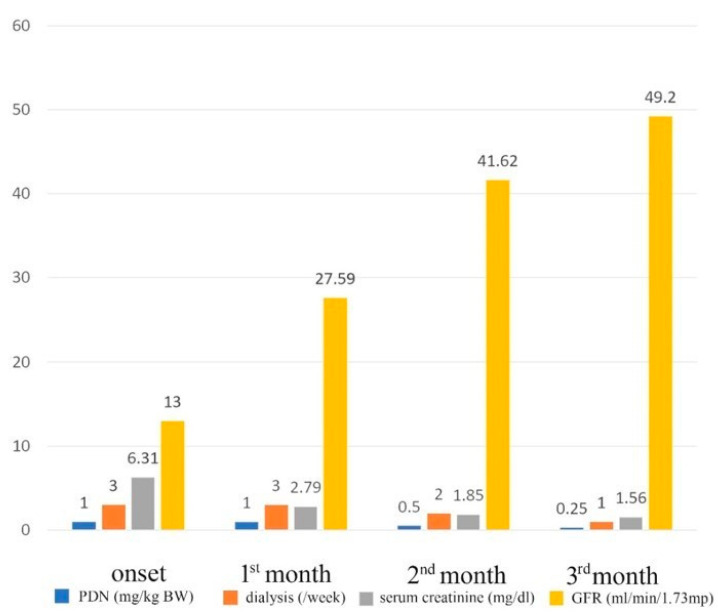
Characteristics of our patient at presentation, 1, 2 and 3 months after therapy initiation.

Disease activity can be followed by angiotensin-converting enzyme (ACE) serum levels. We note an improvement of this parameter 3 months after cortisone therapy as well as an overall improvement of renal function, with dialysis discontinuation and hepatosplenomegaly diminution (Figure 10).

Our evaluation concluded with the diagnosis of extrapulmonary pediatric-onset adult-type sarcoidosis with nephrocalcinosis, nephrolithiasis, non-granulomatous nephritis, granulomatous hepatitis, portal hypertension, hepatic insufficiency, splenic infarctions, and band keratopathy.

## 3. Discussions

Sarcoidosis is a disease characterized by the formation of nodules of inflammatory cells or noncaseating granulomas in any part of the body—most commonly in the lungs and lymph nodes. However, it can also affect the eyes, skin, heart, kidneys, central nervous system, or sense organs. Sarcoidosis, or Besnier-Boeck-Schaumann disease, was first identified by the English doctor Johnathan Hutchinson in 1877, and although more than a hundred years have passed since that first description, the disease still remains an enigma [6]. The incidence of sarcoidosis in adults appears to be biphasic [3]. Historically, it was thought to affect young adults between 30 and 50 years, but recent studies have reported that more than half of cases are patients over 55 years of age [3,7]. About 25% of people affected by the disease develop a chronic and progressive disease, which contributes to the increasing disease burden on health systems [8,9].

### 3.1. Pediatric Sarcoidosis—Epidemiology

Since 2008, with the Danish series, around 22 manuscripts on pediatric sarcoidosis have been published annually, compared to over 900 for adult sarcoidosis, with more than half being case reports [10]. Pediatric sarcoidosis is extremely rare, with only three reported cohorts: Danish (Caucasian), French (Afro-Caribbean), and Louisiana (Afro-American) patients. Most patients were aged 11–13 years. The disease seemed severe in children, involving multiple organs, and was often persistent in adulthood [11]. The diagnosis of sarcoidosis is relatively uncommon in children, and the manifestations of the disease may be different in children compared to adults [12]. Sarcoidosis is 3–4 times more common and more aggressive in black than in white patients [13]. Furthermore, the lifetime risk of developing sarcoidosis is higher in African Americans (2.4%) than in whites (0.8%) [14], although the true incidence and prevalence are unknown due to the limited number of reported cases in children. A recent study among Danish patients estimated an incidence of approximately 0.29 per 100,000 person-years in children under 15 years of age. The incidence of pediatric sarcoidosis ranged from 0.06 per 100,000 person-years for children under 5 years of age to 1.02 per 100,000 person-years for children aged 14 to 15 years [15]. Most pediatric cases have been reported in children between 13 and 15 years of age. At the onset, our patient had an age close to the maximum incidence of the disease in children.

### 3.2. Sarcoidosis—Etiopathology

Sarcoid granulomas represent an intensely interconnected network of immune cells, including macrophages, dendritic cells, T helper lymphocytes, T regulatory cells, and their mediators. After years of investigations and advances in medical science, the immunopathogenesis of sarcoidosis remains evasive. One or multiple antigens may trigger an exaggerated cell-mediated immune response resulting in granulomatous inflammation. These antigens, possible etiologic agents, vary from occupational or environmental factors to infectious agents, and, of course, genetic contribution may play an important role [16,17,18]. A multicenter National Institutes of Health (NIH)-funded ACCESS case-control study of over 700 patients and around 30,000 relatives revealed that no single etiologic agent and no genetic locus was clearly implicated in the pathogenesis of sarcoidosis [19]. Recent findings concerning T cell sensitization to aluminum, beryllium, silica, or zirconium in some cases of sarcoidosis suggest the role of triggers in different clinical phenotypes [20]. Several proteins, such as vimentin, a major constituent of the intermediate filament family of proteins expressed in normal mesenchymal cells and involved in maintaining cellular integrity and resistance against stress, along with tubulin and alpha-actinin-4, may induce similar patterns of cytokine secretion by sarcoidosis peripheral blood mononuclear cells [21,22].

Special attention was directed to infectious agents, mostly mycobacteria and cutibacteria (in the past known as propionibacteria). Several studies showed their possible implications for developing sarcoidosis [23,24]. Mycobacterium tuberculosis infection has several histologic similarities with sarcoidosis, which is why this bacterium was intensively evaluated, similar to etiologic causes [25]. In a study using hybridization techniques and polymerase chain reaction, Saboor et al. found evidence of M. tuberculosis in sarcoid tissue, but several years later, Richter et al. established no such findings using similar techniques [26,27]. Infectious agents, such as lymphotropic viruses (HHV6, HHV8, HIV, HTLV1, and cytomegalovirus), have been documented in patients with sarcoidosis but apparently represent generalized B cell activation rather than being a sign of an etiology [23]. Genetic contribution in sarcoidosis was suggested due to the occasional occurrence of sarcoidosis in more than one member of a family [28]. The major histocompatibility complex antigens were most closely linked with genetic susceptibility in sarcoidosis [29]. Genome-wide linkage and some studies have suggested that several genes may be associated with increased susceptibility to sarcoidosis, such as butyrophilin-like 2 gene (*BTNL2*), annexin A11 (ANXA11), and angiotensin-converting enzyme variants, although the associations vary across populations [30,31]. Drug-induced sarcoidosis (antiretroviral therapy, TNF-α antagonists, interferon therapy, and immune checkpoint inhibitors) can only be speculated because of the surprising clinical and histological similarity between the two entities in terms of immunopathogenesis [32].

### 3.3. Sarcoidosis—Laboratory Diagnosis

Sarcoidosis is diagnosed based on clinical and imaging manifestations as well as the histopathological detection of non-caseating granulomas at the level of the affected organs after excluding other diseases. No laboratory marker can be conclusive for the diagnosis of sarcoidosis, although serum angiotensin-converting enzyme is among the most commonly used diagnostic biomarkers for sarcoidosis; however, the test is nonspecific and lacks sensitivity [33]. Regarding sarcoidosis, vitamin D and its metabolism are noteworthy due to their crucial involvement in immune system regulation and granulomatous inflammation. The expression of 1-α-hydroxylase is present in numerous tissues, but only the kidneys, activated macrophages, and placenta have the ability to affect plasma 1,25(OH)2D3 levels through hydroxylation [34]. Hypercalciuria, hypercalcemia, and elevated levels of 1,25-dihydroxy vitamin D (1,25(OH)2D) may also occur in patients with sarcoidosis due to the overproduction of 25-hydroxy vitamin D-1 α-hydroxylase [35,36]. Recent studies involving adult patients have suggested that serum levels of soluble interleukin-2 receptor (sIL-2r) represent a superior and more sensitive biomarker than serum ACE levels in supporting the diagnosis of systemic sarcoidosis [37,38]. It seems that the level of sIL-2r correlates with the level of disease activity and may indicate multisystem involvement [39]. However, most studies of sarcoidosis biomarkers have been conducted only among adult patients. In a 2020 study, Alsarhan et al. discussed the role of sIL-2r levels when combined with serum ACE and 1,25(OH)2D levels in supporting the diagnosis of systemic sarcoidosis in a child, also looking at the trends in the levels of these markers in relation to disease activity and response to treatment [33]. Although various markers, such as adenosine deaminase (ADA) activity, serum amyloid A (SAA) levels, and sIL-2r levels, have been used in adults to support diagnosis and monitor disease activity, none of these markers have been studied widely in children with sarcoidosis. Most studies attest to the fact that ACE and 1,25(OH)2D levels were the most used in juvenile sarcoidosis [3,33,35]. In our patient, the activity of the disease was monitored using serum ACE dosage. We noticed an improvement in this parameter 3 months after the initiation of cortisone therapy, consistent with the improvement in renal function, the interruption of dialysis, and the reduction in hepatosplenomegaly. Furthermore, at the onset, a low value of PTH and 1,25-dihydroxy vitamin D (1,25(OH)3D) was noted in the context of significant hypercalcemia secondary to the inadequate secretion of 1,25-dihydroxy vitamin D (1,25(OH)2D) from the macrophages of sarcoidotic granulomas and a lack of hydroxylation in the kidney. Patients diagnosed with sarcoidosis should be evaluated in terms of kidney function. Renal biopsy remains the gold standard for confirming renal sarcoidosis, although histological lesions are not specific for sarcoidosis, necessitating the exclusion of infection and drug hypersensitivity, which are more common causes of interstitial nephritis [40].

### 3.4. Pediatric Sarcoidosis—Clinical Findings

Regarding the clinical presentation, extrapulmonary manifestations were observed more frequently in symptomatic children and adolescents than in adults [14]. Liver involvement in sarcoidosis is relatively common, with an estimated prevalence of 11.5 to 30% of patients with sarcoidosis [41]. Little is known about the natural course of hepatic sarcoidosis, as most patients remain asymptomatic for a long time. However, some patients may develop severe complications such as cirrhosis and portal hypertension [42,43]. Our patient developed liver damage simultaneously with kidney damage, manifested by portal hypertension syndrome. The liver biopsy performed on admission revealed chronic granulomatous hepatitis with giant cells, which is highly suggestive of sarcoidosis. Very few pediatric series with prolonged follow-ups have been reported. In a series of 52 patients (34 followed prospectively from childhood and 18 retrospectively in adulthood), Chauveau et al., found that the age at onset was 12 (±2.7), and the relapses occurred mainly during the de-escalation of therapy or in the first three years after the interruption of the treatment; rarely is the disease in remission for more than three years. Sarcoidosis was more severe in adulthood [39].

### 3.5. Pediatric Sarcoidosis—Renal Involvement

The reported prevalence of kidney involvement in sarcoidosis varies widely due to the study design and enrolled patient populations, but also due to the heterogeneity and asymptomatic nature of renal disease. Kidney lesions associated with sarcoidosis are represented by specific histological changes in the disease or by abnormal calcium metabolism, nephrolithiasis, and nephrocalcinosis. The renal manifestations in sarcoidosis are represented by hypercalcemia and hypercalciuria, nephrolithiasis and nephrocalcinosis, granulomatous or non-granulomatous interstitial nephritis, glomerular and tubular disease, and ureteral obstruction [44]. A variety of glomerular lesions, including membranous nephropathy, focal segmental sclerosis, mesangioproliferative glomerulonephritis, IgA nephropathy, or crescentic glomerulonephritis, are described as glomerular damage in sarcoidosis, without being able to distinguish it from the primary form of these entities. Tubular dysfunctions can affect the isolated proximal or distal renal tubule, but also in the context of tubulopathy Fanconi syndrome. Polyuria is a common clinical feature, mostly due to hypercalcemia [45]. Our patient had the same types of manifestations at the onset. Initially, we considered our case to be end-stage kidney disease (ESRD) because he had a chronic evolution over one year. First, we thought of a glomerular pathology, such as focal segmental glomerulosclerosis (FSGS), because of proteinuria, hematuria, and kidney failure. The diagnosis of FSGS is exclusively bioptic because there are no distinctive clinical features that differentiate it from other types of chronic glomerulopathy. FSGS in children is often coupled with the risk of progression toward end-stage renal disease [46], similar to our patient, with one-year evolution of hypercalcemia and elevated serum urea and creatinine levels. Thus, we reconsidered our approach because the patient had normal kidney sizes and a good response to corticosteroid therapy, reducing the need for hemodialysis until this therapy was stopped. Therefore, we determined that our patient had acute decompensation of chronic kidney disease (CKD) secondary to sarcoidosis. Prolonged hypercalcemia is responsible for a decrease in the glomerular filtration rate by vasoconstriction of the afferent arteriole. Intracellular calcium overload and tubular obstruction by calcium precipitates lead to acute tubular necrosis. In the absence of adequate treatment, hypercalcemia and hypercalciuria are not reversible, leading to fibrosis, and the damage becomes irreversible [47,48].

Nephrocalcinosis is a result of chronic hypercalciuria and is a rare finding in sarcoidosis [49]. It is present in less than 5% of patients with sarcoidosis but in a higher rate of patients with renal insufficiency [50]. Nephrocalcinosis is a significant cause of chronic kidney disease [51]. Hypercalcemic nephropathy is the main cause that leads to ESRD in renal sarcoidosis, with some cases requiring some form of kidney replacement therapy. Mahevas et al., conducted a study among 46 patients with sarcoidosis-related interstitial nephritis, and only two progressed to ESKD [52]. Nephrolithiasis was reported in about 10% of patients with sarcoidosis, with a prevalence range between 3 and 14% [47]. More recently, Klaus et al., performed a literature review and found a total of 36 cases of renal involvement in pediatric sarcoidosis. The study showed that AKI is a common feature (86%) in pediatric sarcoidosis with renal involvement. Granulomatous interstitial nephritis (GIN) was the predominant histology (66%), and 47% had hypercalcemia. The patients with hypercalcemia showed a trend toward a better prognosis. The negative predictor factors toward CKD are represented by GIN, concentration impairment, and the intensity of GFR impairment at presentation [53].

Coutant et al., retrospectively analyzed 11 children with renal granulomatous sarcoidosis confirmed by renal histology after a mean follow-up of 5.5 years; 3 patients developed end-stage renal failure, and 1 had chronic insufficiency after interruption of medical supervision and prednisone therapy [54]. The evolution to CKD stage 5 was noticed in patients exclusively diagnosed with GIN, while that with CKD 1–2 predominantly had non-granulomatous tubulointerstitial nephritis (ngTIN); all of these findings are supported by data from an adult case series [54]. Patients who have interstitial nephritis may also have nephrolithiasis or nephrocalcinosis [54]. Our patient was evaluated as having improved kidney function after treatment, with his renal histology being nephrocalcinosis, nephrolithiasis, and non-granulomatous nephritis. After multiple debates, the question that nephrocalcinosis and/or GIN might have a better prognosis remains uncertain. In Table S1, we have summarized all the studies of sarcoidosis with renal involvement in children that were the basis of this review [53,54,55,56,57,58,59,60,61,62,63,64,65,66].

### 3.6. Pediatric Extrapulmonary Sarcoidosis—Treatment

Depending on the involvement, extrapulmonary sarcoidosis may benefit from local treatment, escalation of systemic treatment, or multimodal interventions [67]. The majority of studies regarding the treatment of symptomatic sarcoidosis have focused on pulmonary disease [68]. The goals of treatment in sarcoidosis are to prevent permanent end-organ dysfunction, reduce mortality, and preserve the quality of life. Studies in this area are limited. In previously reported cases, the duration of treatment varied from 1 month to 23 years [3,4,5,10,69,70,71]. Treatment should target the granulomatous process (anti-inflammatory drugs) as well as organ-targeted and supportive treatments. The treatment of organ dysfunction can improve the outcome of sarcoidosis and includes non-pharmacological therapies (pacemakers, CSF diversion for hydrocephalus management, and heart, kidney, and liver transplantation) or pharmacological therapies (hormone replacement, antiepileptic drugs, or psychiatric drugs, etc.) [72,73]. Corticosteroids remain the first-line treatment, although they have significant cumulative side effects. Therapies such as alkylating agents, immunosuppressive medication, calcineurin inhibitors, and TNF-α inhibitors can be used in refractory or relapsing diseases or when corticosteroids cause unacceptable toxicity. Methotrexate and infliximab have demonstrated significant efficacy and an acceptable safety profile in cardiac sarcoidosis, for example, [74]. Chronic kidney disease is a contraindication for treatment with methotrexate. Thiopurine S-methyltransferase (TPMT) deficiency is a contraindication for the use of azathioprine [75]. Renal sarcoidosis after kidney transplantation is rare, usually follows a mild clinical course and is responsive to increased immunosuppression. It has been recently reported that it is safe to perform renal transplantation in sarcoidosis with close clinical and histological monitoring [76].

### 3.7. Pediatric Extrapulmonary Sarcoidosis—Evolution

Advanced sarcoidosis refers to manifestations of the disease with high mortality (pulmonary fibrosis, pulmonary hypertension, cardiac sarcoidosis, renal sarcoidosis, and neurosarcoidosis) and requires treatment in the stage of disease activity, with long-term maintenance therapy being controversial [77]. Treatment protocols for pediatric renal sarcoidosis are not yet well established. In all reported cases, prednisolone was the standard treatment. Several immunosuppressants, including mycophenolate mofetil, cyclophosphamide, and methotrexate, were added in refractory cases or to spare steroids, and also TNF-α inhibitors (Infliximab) were used after steroid-resistant/refractory disease [78,79]. The outcome of kidney transplantation is not well known, and the recurrence of renal sarcoidosis has been described in adults [80,81]. For children, the outcome is variable for sarcoidosis, ranging from spontaneous remission to end-stage renal disease. Early diagnosis and prompt treatment with corticosteroids can improve prognosis [82]. Hypercalcemia can be responsible for acute kidney injury (AKI) caused by vasoconstriction of afferent arterioles [66], as in the case presented, but which, in the context of persistent hypercalcemia, was complicated by nephrocalcinosis. In patients with ESRD, dialysis and transplantation can provide results comparable to those observed in patients with other causes of renal failure.

### 3.8. Pediatric Extrapulmonary Sarcoidosis—Prognosis

The prognosis of the disease is generally good, with the exception of patients with severe cerebral deterioration or those with frequent relapses [83]. The presence of hydrocephalus implies a mortality of up to 75% [84]. Classically, the prognosis of sarcoidosis in children depends on the age of onset, the extent of the disease, and the response to corticosteroids. However, the prognostic factors still remain unclear in children [70]. In children, studies on the natural history of sarcoidosis are rare. In the Danish cohort with a median follow-up of 15 years, 78% of patients recovered completely, 11% still had active disease, 4% had remission with organ involvement, and 7% died [4,71,85]. Being a complex systemic disease, sarcoidosis can present itself with a wide range of signs and symptoms, being often considered one of the important “imitators” in pathology. In this context, a multidisciplinary approach to the patient is necessary. Early identification and establishment of the therapeutic strategy influences the morbidity and mortality of the disease. Mortality in sarcoidosis is affected by factors such as age, gender, race, organ involvement, care, and treatment and is estimated to be 1–8% [86]. The treatment of sarcoidosis can also lead to complications (infections, malignancy), which can influence the prognosis of the disease. Immunosuppressive drugs have been shown to increase the risk of infection in sarcoidosis [87]. In the literature, the relationship between sarcoidosis and malignancy is well known. According to the results of various studies, sarcoidosis has been considered a risk factor for the development of cancer, especially lymphoproliferative cancer [88]. In general, diseases can be classified according to their activity or severity, but in sarcoidosis, disease activity does not necessarily indicate a fatal prognosis or the need for treatment. This is a challenge for the clinician, who faces many difficulties when trying to classify late-onset pediatric sarcoidosis.

## 4. Conclusions

Sarcoidosis with extrapulmonary involvement in children is rare, which leads to a delayed diagnosis. In our case, renal sarcoidosis was diagnosed in advanced CKD, with severe GFR impairment at onset, offering unfavorable outcomes. Renal biopsy is mandatory in this case for a better understanding of the disease course and reassessment of the treatment. Sarcoidosis cannot necessarily be considered a benign nephrologic condition and should be considered in the differential diagnosis of pediatric patients with AKI/CKD of an unknown etiology. Oral or pulses of intravenous corticosteroids are the mainstay of the treatment of sarcoidosis in children. Relapses are frequently observed in the evolution of this pathology, mainly extrathoracic, so even in cases of initial remission, long-term follow-up is required. There are some clinical, radiological, and laboratory factors that determine the prognosis of the disease. The clinical phenotype of the disease can be presented with self-limited acute sarcoidosis and/or chronic, progressive multisystemic involvement. Laboratory factors are nonspecific and may not always be useful in clinical practice. New biomarkers would be useful for accurate diagnosis. New multicenter prospective studies are needed to shed light on this pathology, especially in children.

## Figures and Tables

**Table 1:** Clinical, biological, and histopathological parameters at onset and 3 months after prednisolone therapy.

	Onset	After 3 Months
**General symptoms**	Fatigue, HSM	Cushing syndrome, no HSM
**Laboratory**		
TGP	71 U/L	34 U/L
TGO	86 U/L	25 U/L
Alkaline phosphatase (ALP)	286 U/L	213 U/L
GGT	163 U/L	54 U/L
CRP	28.49 mg/dL	4 mg/dL
ESR	61 mm/1 h	10 mm/1 h
ACE	105 U/L	20 U/L
Calcium	12.9 mg/dL	9.8 mg/dL
**Nephrological**		
Serum creatinine	6.31 mg/dL	1.56 mg/dL
eGFR (Schwartz’s pediatric equation)	13 (mL/min/1.73 m^2^) before dialysis initiation	49.2 mL/min/1.73 m^2^ dialysis discontinuation
Hematuria	+++	+
Proteinuria/24 h	848 mg/day	627 mg/day
Leukocyturia	++	+
Urinary Calcium/creatinine ratio	0.7	0.21
Urinary 24 h calcium	5.19 mg/kg/day	2.9 mg/kg/day
B2M	437 mcg/L	325 mcg/L
Hemodialysis	3 times/week	Dialysis discontinuation
**Hepatic histology**	Granulomatous aspect	Not repeated
**Ophthalmology**	Band keratopathy	Not repeated

HSM = hepatosplenomegaly; HD = hemodialysis; TGP = transaminase glutâmico pirúvica; TGO = transaminase glutâmico oxalacética; GGT = gamma-glutamyl transferase; CRP = C-reactive protein; ESR = erythrocytes sedimentation rate; ACE = angiotensin-converting enzyme; eGFR = estimated glomerular filtration rate; B2M = urinary beta 2 microglobulin.

## Data Availability

All medical records are from Hospital Info Word application of our institution and can be accessed with the manager approval.

## Supplementary Materials

The following supporting information can be downloaded at: https://www.mdpi.com/article/10.3390/ijms24087327/s1.
