# Supplementary material for: The Overlap of Kidney Failure in Extrapulmonary Sarcoidosis in Children—Case Report and Review of Literature
Source: Int J Mol Sci. 2023 Apr 15;24(8):7327. doi: 10.3390/ijms24087327 (PMC10138650; doi:10.3390/ijms24087327)
Supplement: Supplementary file 1 [file ijms-24-07327-s001.zip › ijms-2289468-supplementary.pdf]

Authors, year of publication	Study design	Age at diagnosis /sex	Type of pediatric Sarcoidosis	Renal involvement	Other extrapulmonary involvement	Pulmonary involvement	Treatment	Follow Up
**Klaus *et al.*, 2021 [54]**	1 case	16 year-old / caucasian female	pediatric-onset adult-type sarcoidosis	Granulomatous interstitial nephritis<br>AKI	Granulomatous iridocyclitis<br>Giant-cell sialadenitis<br>Arthralgia	yes	MTP 300 mg/m² intravenously on days 1, 3, and 5 with 80 mg PDN orally on days 2, 4, and 6. From day 7, oral PDN was continued with 60 mg daily.<br>Oral MTX (20 mg weekly) for multiorgan involvement. PDN was slowly tapered, aiming for a 6-month period to achieve the maintenance dose of 5 mg daily.	Follow up 7 months<br>One month after therapy initiation, kidney function normalized - GFR, 108 ml/min/1.73
**Coutant *et al.*, 1999 [55]**	11 case series	Median age at onset 10.1 years, 6 male/5 female	pediatric-onset adult-type sarcoidosis	Glomerular fibrosis 4/11<br>AKI 9/11	Eye 6/11<br>Arthritis 3/11<br>Liver 7/11 Spleen 5/11	yes, 6/11	Oral PDN (1 mg/kg body weight per day)	Follow up 5,5 years<br>CKD 2/11<br>ESRD 2/11<br>GFR >80 ml/min/1.73 m² -7/11
**Fink *et al.*, 1997 [56]**	6 case series	Less than 4 years, 5 whites male and one Latin-American male	early onset type	Transitory AKI	Rash<br>Arthritis<br>Uveitis	1 patient	MTP pulses, oral PDN (1 mg/kg body weight per day)	Long followup (9-23 years), severe complications: blindness (4 patients), growth retardation (3 patients), heart involvement (2 patients), renal failure (1 patient), death in one patient.
**Dimitriades *et al.*, 1999 [57]**	1 case	13 year-old / caucasian female	pediatric-onset adult-type sarcoidosis	Membranous nephropathy manifested by Nephrotic syndrome	Fever<br>Hepato-splenomegaly<br>Arthritis	yes	Oral PDN (1 mg/kg body weight per day) + CYC 6 monthly pulse therapy, than MTX	Follow up 1,5 year<br>Normalized GFR

**Thumfart et al., 2005 [58]**	1 case	13 year-old / caucasian male	pediatric-onset adult-type sarcoidosis	Granulomatous interstitial nephritis	Severe hypertension	no	MTP pulses (3 times, 1 g/m² /d), followed by oral PDN (1 mg/kg body weight per day), 3 months Infliximab (5 mg/kg intravenously every 4 weeks)	4 month follow up - partial recuperation of renal function - GFR > 40 ml/min/1.73 m²
**Moudgil *et al.*, 2006 [59]**	1 case	15 year-old / caucasian male	pediatric-onset adult-type sarcoidosis	Granulomatous interstitial nephritis AKI	Fatigue Weight loss	no	PDN (1 mg/kg body weight per day), 3 months MMF (1g/m²/day)	Follow up 1,8 years
**Rheault *et al.*, 2006 [60]**	1 case	14 year-old / afro-american male	pediatric-onset adult-type sarcoidosis	Granulomatous interstitial nephritis AKI	Hearing loss	no	PDN (1 mg/kg body weight per day), 6 months	Follow up 2 years Did not regain his hearing and received a cochlear implant approximately 9 months after his original presentation Partial regain renal function - GFR 60 ml/min/1.73 m²
**Hobbs *et al.*, 2010 [61]**	1 case		pediatric-onset adult-type sarcoidosis	Granulomatous interstitial nephritis	no	no	?	?
**Ito *et al.*, 2009 [62]**	1 case	17 year-old / japanese male	pediatric-onset adult-type sarcoidosis	Granulomatous interstitial nephritis AKI	Fever Abdominal pain, diarheea Chorioretinitis	no	MTP pulses (3 times, 30mg/kg, 3 days), PDN (20mg daily), than, from day 36 Mizoribine 200 mg/day	Follow up 2 years Improve kidney function to 64 ml/min/1,73 m²
**Vargas *et al.*, 2010 [63]**	1 case	7 year-old / afro-american female	pediatric-onset adult-type sarcoidosis	Granulomatous interstitial nephritis with ESRD evolution Recurrence in transplanted kidney	Left eye uveitis Polyarthritis Granulomatous lymphadenitis	no	MTP pulses (3 times, 1 g/m² /d), PDN (1 mg/kg body weight per day), infliximab (5 mg/kg intravenously every 4 weeks),	Follow up 8 years Recurrence after renal transplantation, at age of 15 Died 1 month after recurrence

**Roslinah *et al.*, 2010 [64]**	1 case	7 year-old / asian male	pediatric-onset adult-type sarcoidosis	Granulomatous interstitial nephritis AKI	Uveitis	no	Oral PDN 60mg/day + oral MTX 5mg/day, 3 months,	Follow up 2 year<br>During the tappering dose period, he had an attack of bilateral uveitis, needed topical corticosteroids<br>Regain kidney function after 2 years
**Pepple *et al.*, 2015 [65]**	1case	9 year-old / Caucasian male	pediatric-onset adult-type sarcoidosis	Granulomatous interstitial nephritis	Anterior bilateral uveitis	no	Uveitis: parenteral MTX (25mg/week) and Humira (40 mg/every other week).<br>Interstitial nephritis 40 mg oral prednisone daily	Not mentioned
**Downie *et al.*, 2017 [66]**	1 case			Granulomatous interstitial nephritis AKI	Anterior bilateral uveitis<br>Severe Hypertension	no	Severe hypercalcemia: Calcitonin 130 mg subcutaneously and pamidronate 0.25 mg/kg intravenously (given twice upon improvement of renal function)<br>Renal sarcoidosis: oral PDN 1 year (1 mg/kg body weight per day), MMF 9,5 months (1g/m²/day)	Follow up 1 year: improve GFR to 75 ml/min /1.73 m²
**Wang et al., 2019 [67]**	1 case	10 year-old / chinese male	pediatric-onset adult-type sarcoidosis	Granulomatous interstitial nephritis AKI	Multiple peripheral lymph nodes, liver and spleen with noncaseating granulomas	yes	Oral PDN (2 mg/kg/day) and MMF (1g/m²/day) – 3,5 month, than discontinued MMF and prescribed monthly intravenous pulse therapy with CYC (600 mg/month), 2 months.	Follow up 6 month<br>Improve kidney function to GFR 67.4 ml/min/1.73 m²

Abbreviation: GFR: glomerular filtration rate; CKD: chronic kidney disease; ESRD: end stage renal disease; AKI: acute renal injury; NS: nephrotic syndrome; ACE: angiotensin-converting enzyme; GIN: granulomatous interstitial nephritis; MTX: methotrexate; MTP: Methylprednisolone; PDN: prednisone; MMF: mycophenolate mofetil; CYC: cyclophosphamide
